# Real-world data of the association between quality of life using the EuroQol 5 Dimension 5 Level utility value and adverse events for outpatient cancer chemotherapy

**DOI:** 10.1007/s00520-020-05443-8

**Published:** 2020-04-12

**Authors:** Chiemi Hirose, Hironori Fujii, Hirotoshi Iihara, Masashi Ishihara, Minako Nawa-Nishigaki, Hiroko Kato-Hayashi, Koichi Ohata, Kumiko Sekiya, Mika Kitahora, Nobuhisa Matsuhashi, Takao Takahashi, Kumiko Okuda, Masayo Naruse, Takuma Ishihara, Tadashi Sugiyama, Kazuhiro Yoshida, Akio Suzuki

**Affiliations:** 1 Department of Pharmacy, Gifu University Hospital, Gifu, Japan; 2 Department of Surgical Oncology, Gifu University Graduate School of Medicine, Gifu, Japan; 3 Division of Nursing, Gifu University Hospital, Gifu, Japan; 4 Gifu University Hospital Innovative and Clinical Research Promotion Center, Gifu University, Gifu, Japan; 5 Laboratory of Pharmacy Practice and Social Science, Gifu Pharmaceutical University, Gifu, Japan

**Keywords:** Quality of life, Outpatient cancer chemotherapy, Chemotherapy-induced adverse events, Proportional odds logistic regression model, Retrospective descriptive study

## Abstract

**Background:**

Outpatient cancer chemotherapy may lead to improved quality of life (QOL) by allowing treatment to continue without impairing the social lives of patients compared with hospitalization. However, the occurrence of serious adverse events may cause a decline in QOL. We investigated the relationship between outpatient chemotherapy–induced adverse events and QOL.

**Methods:**

A single-center retrospective descriptive study was conducted in patients who received outpatient chemotherapy at Gifu University Hospital (Gifu, Japan) between September 2017 and December 2018. The utility values of QOL, type and severity of adverse events, type of cancer, chemotherapy regimen, and other patient demographics were analyzed. Adverse events were graded according to the Common Terminology Criteria for Adverse Events, version 4.0. QOL was evaluated using the Japanese version of the EuroQol 5 Dimension 5 Level (EQ-5D-5L). Associations between the EQ-5D-5L utility value and serious adverse events were assessed using adjusted (age and sex) odds ratios obtained with a proportional odds logistic regression model.

**Results:**

Data from 1008 patients who received 4695 chemotherapy cycles were analyzed. According to proportional odds logistic regression, the adverse events that significantly correlated with a decreased EQ-5D-5L utility value were malaise, edema of the limbs, peripheral neuropathy, pruritus, and dry skin. Based on the proportional odds logistic analysis, neither cancer type nor anticancer drugs were significantly correlated with the EQ-5D-5L utility value in patients who received chemotherapy. Pharmaceutical care for peripheral neuropathy significantly improved patients’ EQ-5D-5L utility value from 0.747 to 0.776 (*P* < 0.01).

**Conclusions:**

Adverse events (i.e., peripheral neuropathy, malaise, and edema of the limbs) are significantly correlated with a decrease in QOL, regardless of the type of cancer or anticancer drugs used. Pharmaceutical care provided by pharmacists in collaboration with physicians may improve QOL.

**Electronic supplementary material:**

The online version of this article (10.1007/s00520-020-05443-8) contains supplementary material, which is available to authorized users.

## Introduction

The number of patients who undergo cancer chemotherapy is increasing in parallel with the morbidity and mortality associated with cancer worldwide. Moreover, cancer chemotherapy has transitioned from inpatient to outpatient settings because of advancements in supportive care measures against cancer and changes in the healthcare environment to reduce medical costs [1–4]. Hence, patients are able to continue their personal life and work by undergoing chemotherapy in an outpatient setting. In fact, Ishiura et al. [5] reported that in patients with non-small lung cancer who received vinorelbine, “psychological condition” related to quality of life (QOL) was significantly improved by a transition from inpatient therapy to outpatient chemotherapy.

However, outpatient cancer chemotherapy is characterized by a high incidence of adverse events [6–8], and severe adverse events may directly influence the personal life and work of patients. This effect may reduce patients’ QOL. Tachi et al. [8] showed that the occurrence of anorexia induced by chemotherapy significantly reduced the QOL of patients with breast cancer. Furthermore, Mark et al. [9] reported that patients with advanced-stage lung cancer who experienced strong negative feelings related to side effects have decreased health-related QOL, and recommended facilitating vigorous management of low-grade adverse events to enhance the health-related QOL of patients. Moreover, Hagiwara et al. [10] showed that grade 1 oral mucositis, grade 1 and 2 fatigue, and grade 2 sensory neuropathy were significantly associated with impaired global health status in the European Organization for Research and Treatment of Cancer Quality of Life Questionnaire Version 3.0 in patients receiving first-line chemotherapy for metastatic breast cancer.

These findings highlight the importance of reducing adverse events in order to maintain QOL in patients receiving outpatient chemotherapy. Nevertheless, these studies [8–10] investigated only a limited number of cancer types and anticancer agents. Few cross-sectional studies have investigated the association between a decline in QOL and adverse events in patients with a variety of cancer types and taking a variety of anticancer drugs.

In this study, we conducted a retrospective analysis to investigate the impact of current outpatient chemotherapy-related adverse events on QOL.

## Patients and methods

### Study design

This single-center, retrospective, and descriptive study was conducted at Gifu University Hospital, which is affiliated with Gifu University (Gifu, Japan). Patients who underwent cancer chemotherapy at the Gifu University Hospital outpatient cancer chemotherapy clinic between September 2017 and December 2018 were enrolled in the present study. The utility values of QOL, type and severity of adverse events, type of cancer, chemotherapy regimen, and other patient demographics were extracted from the electronic medical records of the hospital and retrospectively analyzed.

### Outpatient chemotherapy clinic

We previously reported the system in our outpatient chemotherapy clinic [11, 12]. Briefly, full-time medical staff consisted of two physicians, eight nurses, and four pharmacists. The pharmacists verified prescription orders based on patients’ cancer chemotherapy regimens, provided pharmaceutical care services to all outpatients who received cancer chemotherapy, monitored adverse events, and proposed prescriptions to physicians regarding supportive care. The pharmacists also provided drug information to other medical staff.

### Assessment of QOL

The EuroQol 5 Dimension 5 Level (EQ-5D-5L) questionnaire was developed by the EuroQol group to investigate health-related QOL in adults [13]. The Japanese version of the EQ-5D-5L was developed by Shiroiwa et al. [14] to evaluate QOL reflecting Japanese values. The EQ-5D-5L is widely used in clinical studies and health status surveys targeting the general population and uses a comprehensive scale based on preferences to assess cardinal changes in health status [15, 16]. While the values in the Japanese version [14] differ from those in the original, the utility values of QOL in this study were calculated using the Japanese version of the EQ-5D-5L to reflect the values of the Japanese people. We used a hybrid model prepared by mapping discrete choice experiment (DCE) data onto composite time trade-off (cTTO) data [14] to determine the EQ-5D-5L utility value.

We applied to the EuroQoL Group for use of the Japanese version of the questionnaire and obtained permission before use. The Japanese version of the EQ-5D-5L questionnaire was used in face-to-face interviews to estimate the utility values of QOL [14] and was routinely implemented by pharmacists during each patient visit. The utility values were recorded in the hospital’s electronic medical records.

The five dimensions assessed by the EQ-5D-5L are mobility, self-care, usual activities, pain/discomfort, and anxiety/depression. Each of these is assessed according to five levels of severity: level 1, no problem; level 2, slight problem; level 3, moderate problem; level 4, severe problem; and level 5, unable or extreme problem [13]. A utility value ranging from 0 to 1 was calculated from the EQ-5D-5L, which was defined as the primary outcome of this study. According to the Japanese version of the utility value conversion table, “0” indicates death and “1” indicates full health [17]. The EQ-5D-5L contains only five questions, and patients receiving outpatient chemotherapy can easily answer these questions at each cycle. For these reasons, we used the EQ-5D-5L questionnaire in the present study.

### Assessment of adverse events

All patients were provided with a daily checklist to confirm their side effects on their first visit to the outpatient chemotherapy clinic. Using the checklist, patients recorded the occurrence of daily adverse events after chemotherapy. From the returned checklists and the results of the interviews, pharmacists, in collaboration with physicians, recorded the severity of adverse events in the electronic medical records. The severity of adverse events was graded according to the Common Terminology Criteria for Adverse Events version 4.0 (National Cancer Institute, Bethesda, MD, USA) [18].

If moderate or severe adverse events occurred in a patient receiving outpatient chemotherapy, physicians and pharmacists implemented a pharmaceutical care intervention based on clinical practice guidelines. Pharmaceutical care for adverse events was provided by pharmacists in collaboration with physicians, and the impact of this intervention on the adverse events was assessed during the subsequent visit.

### Effect of pharmaceutical care on peripheral sensory neuropathy

Evidence suggests that moderate peripheral neuropathy (grade ≥ 2) has a strong negative impact on QOL [10]. Therefore, we investigated the changes in QOL induced by anticancer drugs in patients with peripheral neuropathy. These changes were examined at three time points: prior to peripheral sensory neuropathy (control), during the development of peripheral sensory neuropathy (pre-intervention), and after pharmaceutical intervention for peripheral sensory neuropathy (post-intervention).

Specifically, to show that utility values for QOL were reduced by the appearance of peripheral neuropathy, we compared utility values for QOL between “control” and “pre-intervention” time points. In addition, to show that the effect of pharmaceutical intervention for peripheral neuropathy increased utility values for QOL, we compared utility values for QOL between “pre-intervention” and “post-intervention” time points.

### Statistical analysis

Patient demographics were summarized using medians with the 25th and 75th percentiles for parametric variables. Frequencies and percentages are shown for non-parametric variables. As the distribution of the EQ-5D-5L utility value was heavily skewed, we employed proportional odds logistic regression to assess the effect of adverse events on QOL after adjusting for covariates. The proportional odds logistic model, also termed the ordinal logistic model, is a popular model for analyzing ordered outcome variables. This model performs well for skewed continuous outcome variables using the ranks of data. In addition to the moving difference between the current and previous grade of adverse events (changing grade), age and sex were included in the multivariable model. Adjusted associations were analyzed using a regression model with the Huber-White robust sandwich estimator, with patients as a clustering variable. In the secondary analysis, we confirmed the effect of cancer type and anticancer drugs on the EQ-5D-5L utility value using a proportional odds logistic model with adjustment for covariates. An adjusted odds ratio < 1 indicates that QOL is more likely to be worse on average in patients with adverse events, cancer, or those taking anticancer drugs. For comparisons assessing the effects of pharmaceutical intervention on peripheral neuropathy, the Wilcoxon signed-rank test for pair-wise comparisons was performed.

Findings with two-sided *P* values < 0.05 were considered statistically significant. Data were analyzed using IBM SPSS version 22.0 (IBM Japan Ltd., Tokyo, Japan) and *R* software version 3.5.1 (www.r-project.org).

### Ethical considerations

The present study was performed in accordance with the guidelines for care in human studies adopted by the Medical Review Board of Gifu University Graduate School of Medicine, and was approved by the Institutional Review Board of the Japanese Government (approval no. 2019-004). Owing to the retrospective nature of the study, the provision of informed consent by the patients was not required.

## Results

### Patients

Patient demographics are shown in Table 1. A total of 1008 patients received 4695 chemotherapy cycles between September 2017 and December 2018 in our outpatient chemotherapy clinic. The most common type of cancer was colorectal cancer (16.8%), followed by gastric cancer (15.1%), lung cancer (12.1%), breast cancer (11.5%), malignant lymphoma (6.7%), pancreatic cancer (6.4%), head and neck cancer (4.1%), and esophageal cancer (1.7%).

**Table 1:** Patient demographics

Number of patients (male/female)	1008	(516/492)
Age, median (min–max)	67	(18–90)
Number of chemotherapy courses	4695	
Cancer		
Colorectal cancer	169	16.8%
Gastric cancer	152	15.1%
Lung cancer	122	12.1%
Breast cancer	116	11.5%
Ovarian cancer/cervical cancer/uterine cancer	113	11.2%
Malignant lymphoma	68	6.7%
Pancreatic cancer	65	6.4%
Bladder cancer/testicular cancer/urothelial cancer	57	5.7%
Head and neck cancer	41	4.1%
Leukemia	26	2.6%
Biliary tract cancer	19	1.9%
Esophageal cancer	17	1.7%
Malignant melanoma	16	1.6%
Malignant soft tissue tumor	12	1.2%
Malignant glioma	12	1.2%
Neuroendocrine carcinoma	3	0.3%
Regimen		
L-OHP + fluoropyrimidines ± Bmab/Cmab/Pmab	154	15.3%
Weekly PTX/Nab-PTX ± Tmab/ramucirumab/Cmab	132	13.1%
Pembrolizumab/nivolumab	115	11.4%
CBDCA + PTX/PEM/DOC/VNR/GEM/S-1/CPT-11 ± Bmab	87	8.6%
Maintenance chemotherapy (Bmab/Tmab/rituximab)	76	7.5%
CPT-11 ± fluoropyrimidines ± Bmab/aflibercept/ramucirumab	57	5.7%
GEM ± S-1	38	3.8%
DOC/GEM/LipoDOX/VNR ± Bmab	35	3.5%
FOLFIRINOX/FOLFOXIRI ± Bmab/Cmab	30	3.0%
GEM + Nab-PTX	28	2.8%
CHOP/THP-COP ± rituximab	28	2.8%
PEM ± Bmab	26	2.6%
DOC/GEM/EPI/VNR/S-1 ± PER ± Tmab	25	2.5%
Anthracyclines + cyclophosphamide	25	2.5%
Fluoropyrimidines/TAS102 + Bmab	22	2.2%
S-1 + DOC	14	1.4%
Rituximab + bendamustine	13	1.3%
Cisplatin + GEM	9	0.9%
Cmab/Pmab	9	0.9%
Other	85	8.4%

*L-OHP*, oxaliplatin; *Bmab*, bevacizumab; *Cmab*, cetuximab; *Pmab*, panitumumab; *PTX*, paclitaxel; *Nab-PTX*, nanoparticle albumin-bound paclitaxel; *Tmab*, trastuzumab; *CBDCA*, carboplatin; *PEM*, pemetrexed; *DOC*, docetaxel; *VNR*, vinorelbine; *GEM*, gemcitabine; *S-1*, tegafur + gimeracil + oteracil; *CPT-11*, irinotecan; *LipoDOX*, doxorubicin liposomal; *EPI*, epirubicin; *FOLFIRINOX/FOLFOXIRI*, L-OHP + CPT-11 + 5-FU; *CHOP*, cyclophosphamide + doxorubicin + vincristine + prednisolone; *THP-COP*, cyclophosphamide + pirarubicin + vincristine + prednisolone; *TAS102*, trifluridine + tipiracil

The most common type of regimen was oxaliplatin-based chemotherapy (15.3%), followed by paclitaxel/nanoparticle albumin-bound paclitaxel-based chemotherapy (13.1%), and pembrolizumab/nivolumab (11.4%).

### Relationship between the degree of changing grade for adverse events and the EQ-5D-5L utility value

The mean EQ-5D-5L utility value of all enrolled patients was 0.827. The mean EQ-5D-5L utility value for each patient visit is shown in Supplemental Table 1 by cancer type, regimen, and adverse events. The mean EQ-5D-5L utility value when any adverse event occurred was lower than that of all patients.

Incidence of adverse events (grade ≥ 2) in patients under different regimens is shown in Table 2. Although the incidence of constipation (grade ≥ 2) was more than 10% higher in patients receiving vincristine, no other adverse events occurred at ≥ 10% incidence for any given regimen.

**Table 2:** Incidence of grade ≥ 2 adverse events in patients under different regimens

Adverse event	Oxaliplatin	Paclitaxel	Irinotecan	Cetuximab/panitumumab	Anthracycline + cyclophosphamide	Vincristine	Docetaxel	Carboplatin	Gemcitabine	Nivolumab/pembrolizumab
	*N* = 726	*N* = 1259	*N* = 529	*N* = 221	*N* = 181	*N* = 113	*N* = 172	*N* = 188	*N* = 547	*N* = 568
Constipation	2.75%	3.57%	2.08%	0.04%	9.39%	15.04%	7.56%	3.72%	3.47%	1.41%
Nausea	2.62%	0.48%	3.78%	0.01%	1.66%	0.00%	0.58%	0.00%	0.91%	0.18%
Diarrhea	0.55%	0.56%	0.57%	0.00%	0.00%	0.00%	0.00%	0.00%	0.18%	0.53%
Vomiting	0.41%	0.32%	0.57%	0.00%	0.55%	0.00%	0.00%	0.00%	0.37%	0.35%
Oral mucositis	0.55%	0.08%	1.13%	0.01%	1.66%	0.88%	2.33%	0.00%	0.00%	0.00%
Malaise	1.52%	2.07%	1.89%	0.01%	2.76%	4.42%	2.91%	4.26%	4.20%	0.53%
Pain	0.00%	1.03%	0.00%	0.00%	0.00%	0.00%	0.00%	0.00%	0.73%	0.00%
Edema limbs	0.00%	0.08%	0.00%	0.00%	0.00%	0.00%	1.16%	0.00%	0.00%	0.88%
Nail fever	0.69%	0.00%	0.76%	0.00%	0.00%	0.00%	0.00%	0.00%	0.00%	0.00%
Anorexia	3.72%	2.94%	3.78%	0.00%	0.55%	0.00%	7.56%	2.66%	3.29%	2.11%
Arthralgia	0.00%	0.64%	0.00%	0.00%	0.00%	0.00%	0.00%	0.53%	0.73%	0.00%
Muscle pain	0.00%	0.56%	0.00%	0.00%	0.00%	0.00%	0.00%	0.53%	0.91%	0.00%
Tumor pain	0.41%	0.87%	0.57%	0.00%	0.00%	0.00%	0.58%	0.00%	0.37%	0.70%
Peripheral neuropathy	3.17%	3.65%	3.78%	0.03%	6.08%	9.73%	0.58%	0.00%	3.29%	1.23%
Taste disorder	0.96%	0.87%	0.95%	0.00%	0.55%	0.00%	2.33%	0.00%	3.66%	0.53%
Hand-foot syndrome	0.14%	0.00%	0.00%	0.00%	0.00%	0.00%	0.00%	0.00%	0.00%	0.00%
Alopecia	0.14%	5.00%	0.95%	0.00%	0.55%	0.00%	4.07%	2.66%	2.01%	1.06%
Pruritus	0.00%	0.00%	0.19%	0.00%	0.00%	0.00%	0.58%	0.00%	0.00%	0.00%
Dry skin	0.41%	0.00%	0.95%	0.00%	0.00%	0.00%	1.16%	0.00%	0.00%	0.18%
Acneiform eruption	0.14%	0.08%	0.19%	0.00%	0.00%	0.00%	0.00%	0.00%	0.00%	0.00%

We analyzed the relationship between the degree of changing grade for adverse events and the EQ-5D-5L utility value using proportional odds logistic regression. As shown in Table 3, malaise, edema of the limbs, peripheral sensory neuropathy, pruritus, and dry skin were significantly correlated with a decreased EQ-5D-5L utility value (malaise: odds ratio [OR] 0.18, *P* = 0.001; edema of the limbs: OR 0.09, *P* = 0.031; peripheral sensory neuropathy: OR 0.1, *P* < 0.001; pruritus: OR 0.14, *P* = 0.001; dry skin: OR 0.05, *P* = 0.01).

**Table 3:** Multivariable proportional odds logistic analysis of adverse events (A), cancer type (B), and anticancer drugs (C) and their association with the EQ-5D-5L utility value in patients who received chemotherapy

Factor	Adjusted odds ratio	95% CI	*P* value
A: adverse events			
Constipation	0.55	0.23–1.34	0.191
Nausea	4.37	0.83–23.02	0.082
Diarrhea	1.66	0.18–15.58	0.659
Vomiting	0.01	0–3.03	0.121
Oral mucositis	1.33	0.25–7.17	0.741
Malaise	0.18	0.06–0.5	0.001
Pain	1.19	0.22–6.4	0.836
Edema of the limbs	0.09	0.01–0.8	0.031
Nail fever	7.5	1.27–44.23	0.026
Anorexia	1.44	0.32–6.54	0.639
Arthralgia	1.6	0.25–10.1	0.618
Muscle pain	0.95	0.09–9.91	0.968
Tumor pain	0.14	0–8.89	0.355
Peripheral neuropathy	0.1	0.03–0.35	< 0.001
Taste disorder	0.68	0.21–2.21	0.518
Hand-foot syndrome	0.77	0.14–4.15	0.762
Alopecia	2.03	0.76–5.43	0.157
Pruritus	0.14	0.04–0.43	0.001
Dry skin	0.05	0.01–0.49	0.01
Acneiform eruption	0.21	0.01–3.06	0.255
B: cancer type			
Pancreatic cancer	0.64	0.35–1.15	0.134
Gastric cancer	0.76	0.44–1.32	0.324
Esophageal cancer	1.45	0.7–3	0.318
Head and neck cancer	0.78	0.37–1.66	0.524
Colorectal cancer	0.96	0.64–1.45	0.858
Lung cancer	0.99	0.63–1.53	0.951
C: anticancer drugs			
Oxaliplatin	0.96	0.68–1.35	0.811
Paclitaxel	0.91	0.64–1.3	0.623
Irinotecan	0.71	0.47–1.05	0.088
Cetuximab/panitumumab	1.1	0.6–2.02	0.76
Anthracycline + cyclophosphamide	1.7	0.94–3.07	0.081
Vincristine	0.47	0.19–1.14	0.093
Docetaxel	1.2	0.6–2.41	0.612
Carboplatin	0.86	0.56–1.34	0.511
Gemcitabine	0.84	0.46–1.54	0.568
Nivolumab/pembrolizumab	0.9	0.57–1.45	0.675

Adjusted odds ratio (OR) and 95% confidence intervals (CI) are indicated. Analysis was performed with adjustment for age, sex, and administered cycle

### Assessment of QOL using the EQ-5D-5L utility value

We investigated the association between the EQ-5D-5L utility value and cancer type, including pancreatic cancer, gastric cancer, esophageal cancer, head and neck cancer, colorectal cancer, and lung cancer. We performed proportional odds logistic analysis, adjusting for age and sex. Breast and gynecologic cancers, in which all patients are female, could not be adjusted for sex. This analysis excluded these cancer types and was limited to cancer types that affect both male and female patients. As shown in Table 3, based on the proportional odds logistic analysis, cancer type was not significantly correlated with the EQ-5D-5L utility value in patients who received chemotherapy. However, patients with pancreatic cancer showed a tendency toward reduced EQ-5D-5L utility values (OR 0.64, *P* = 0.134).

In addition, we investigated the association between the EQ-5D-5L utility value and anticancer drugs, such as oxaliplatin, paclitaxel, irinotecan, cetuximab/panitumumab, anthracycline plus cyclophosphamide, vincristine, docetaxel, carboplatin, gemcitabine, and nivolumab/pembrolizumab. As shown in Table 3, administration of irinotecan and vincristine tended to decrease the EQ-5D-5L utility value (irinotecan: OR 0.71, *P* = 0.088; vincristine: OR 0.47, *P* = 0.093).

### Change in the EQ-5D-5L utility value after pharmaceutical care for peripheral sensory neuropathy

Of the 163 patients who experienced peripheral sensory neuropathy, 36 patients underwent pharmaceutical intervention for peripheral neuropathy. The demographics of the patients are shown in Supplemental Table 2. We evaluated the degree of change in the EQ-5D-5L utility value for the control, pre-intervention, and post-intervention time points.

The compositions of the regimens were as follows: containing taxane (17 patients, 47.2%), containing oxaliplatin (14 patients, 38.9%), and others (five patients, 13.9%). Details of the pharmaceutical interventions were the additional oral administration of pregabalin (36.1%), duloxetine (55.6%), and gosyajinkigan (2.8%). The latter is a Japanese herbal medicine used to alleviate neuropathy and general pain. Two patients underwent cryotherapy for the hands and feet during the administration of anticancer drugs.

**Fig. 1:**
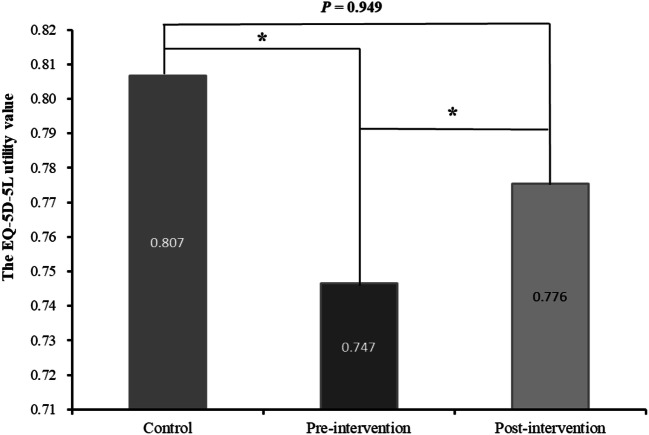
Comparison of mean EuroQol 5 Dimension 5 Level utility values among the control, pre-intervention, and post-intervention time points for peripheral neuropathy. The Wilcoxon signed-rank test was used. Asterisk indicates *P* < 0.05. Control, prior to the development of peripheral sensory neuropathy: pre-intervention, during the development of peripheral sensory neuropathy; post-intervention, after pharmaceutical intervention for peripheral sensory neuropathy

As shown in Fig. 1, the EQ-5D-5L utility value was significantly decreased after the development of peripheral sensory neuropathy (control, 0.807; pre-intervention, 0.747; *P* < 0.001), and significantly higher after pharmaceutical intervention (pre-intervention, 0.747; post intervention, 0.776; *P* = 0.015).

As shown in Table 4, among the five dimensions, the score for mobility, personal care, pain/discomfort, and anxiety/depression was improved by pharmaceutical intervention. In particular, the change noted in the pain/discomfort score was the most pronounced.

**Table 4:** The 5 dimensions of the EuroQol 5 Dimension 5 Level questionnaire at control, pre-intervention, and post-intervention time points for peripheral neuropathy

		Control	Pre-intervention	Post-intervention
Mobility	1	57%	33%	36%
	2	43%	58%	56%
	3	0%	6%	8%
	4	0%	3%	0%
	5	0%	0%	0%
Personal care	1	86%	67%	81%
	2	14%	28%	14%
	3	0%	6%	6%
	4	0%	0%	0%
	5	0%	0%	0%
Usual activities	1	39%	44%	44%
	2	54%	47%	44%
	3	4%	8%	8%
	4	4%	0%	3%
	5	0%	0%	0%
Pain/discomfort	1	50%	19%	33%
	2	46%	53%	56%
	3	4%	22%	6%
	4	0%	6%	6%
	5	0%	0%	0%
Anxiety/depression	1	54%	42%	56%
	2	43%	50%	36%
	3	4%	6%	8%
	4	0%	3%	0%
	5	0%	0%	0%

The values for the 5 dimensions indicate percentage of each item

## Discussion

In this study, we used real-world data to examine the potential association between QOL using the EQ-5D-5L and adverse events in 1008 patients who received 4695 cycles of outpatient cancer chemotherapy. Clinical trial data were obtained from a “selected population” that met the inclusion and exclusion criteria defined in the protocol. Additionally, real-world data were obtained from daily medical practice. Therefore, the data were not limited to the background of patients but are considered real-world data as they represent the actual situation in clinical practice.

Multivariable proportional odds logistic analysis indicated that malaise, edema of the limbs, peripheral sensory neuropathy, pruritus, and dry skin were significant factors for reducing the EQ-5D-5L utility value. Several reports support the present results, highlighting the association between adverse events and decreased QOL [8, 19–21]. Secondary analysis using proportional odds logistic regression did not show a significant association between QOL and cancer type or anticancer drugs. Regardless of the type of cancer or anticancer drugs, the development of adverse events appeared to be an important factor for decreasing QOL in patients receiving outpatient chemotherapy.

Tachi et al. [8] showed that in current breast cancer patients, the rate of deterioration of the utility value after treatment was significant for patients with malaise (*P =* 0.028) in the usual activities dimension. Limb edema is a characteristic finding in patients with malnutrition. Onishi et al. [19] reported that malnutrition in cancer patients is associated with decreased QOL. Additionally, Hershman et al., using the Functional Assessment of Cancer Therapy/Gynecologic Oncology Group-Neurotoxicity, reported that QOL scores decreased from 37.5 to 28.7 post-treatment (*P* = 0.0002).

The EQ-5D-5L is not specific to patients with cancer and contains only a few questions; thus, this tool is characterized by low sensitivity. However, this study showed an association between the changing grade for various adverse events and QOL score using the EQ-5D-5L. Although it is important to continuously evaluate the QOL of patients, in the real world, a large number of question items for each relevant anticancer drug can be burdensome to answer and may interfere with continuous evaluation.

Subsequently, we examined changes in the EQ-5D-5L utility value after pharmaceutical care for chemotherapy-induced peripheral neuropathy. Peripheral neuropathy is a relatively frequent adverse event during cancer chemotherapy, including regimens using taxanes, vinca alkaloids, and platinum agents. The occurrence of moderate-to-severe peripheral neuropathy leads to discontinuation of cancer chemotherapy or dose reduction, which reduces dose intensity and the QOL of the patient [10, 22].

The efficacy of the administration of pregabalin [23, 24], duloxetine [25, 26], and gosyajinkigan [27] for improving chemotherapy-induced peripheral neuropathy has been reported. In addition, Hanai et al. [28] reported that cryotherapy is useful for preventing both the objective and subjective symptoms of paclitaxel-induced peripheral neuropathy and resulting dysfunction.

In this study, the EQ-5D-5L utility value was significantly improved after pharmaceutical intervention (pre-intervention: 0.747; post-intervention: 0.776; *P* < 0.01). Although the minimally important difference for Japan reported by McClure et al. [29] in a simulation-based approach was 0.045, the increase in utility value after intervention in our present study did not exceed the difference. However, 0.807 indicates the approximately 45th percentile, and 0.747 indicates the approximately 35th percentile in the overall data. Therefore, given that the change from control to pre-intervention can be interpreted as a decrease in QOL of about 10% in all people, the effect on control from pre-intervention can be considered significant. As with the interpretation above, 0.776 indicates approximately the 41st percentile in the overall data. Thus, the change from pre-intervention to post-intervention contributed to a 6% increase in QOL in the overall data.

Moreover, among the five dimensions (mobility, personal care, usual activities, pain/discomfort, and anxiety/depression), pain/discomfort scores were significantly elevated prior to and after the onset of peripheral neuropathy (from 1.540 to 2.140). Notably, pain/discomfort scores were significantly decreased prior to and after pharmaceutical care (from 2.140 to 1.830). Among the five dimensions, the change in pain/discomfort scores was the most pronounced. This dimension may influence the change in EQ-5D-5L utility values. Costa et al. [30] analyzed the association between the presence of pain and QOL in breast cancer patients using the European Organization for Research and Treatment of Cancer Quality of Life Questionnaire Version 3.0 and showed that pain negatively influenced QOL with or without metastasis. In the present study, pain due to peripheral neuropathy reduced QOL; however, amelioration of pain due to the administration of pregabalin or duloxetine improved QOL. Temel et al. [31] showed that early palliative care for lung cancer patients resulted in improved QOL and extended survival compared with standard care. Improved QOL contributes to prolonging survival; this finding supports the present results showing that pharmaceutical care provided by pharmacists in collaboration with physicians may improve therapeutic efficacy.

There were several limitations to the present study. First, there were no control patients (i.e., individuals who did not receive anticancer drug treatment) for comparison of QOL with that of patients who received cancer chemotherapy. Second, the degree of cancer progression is strongly associated with QOL, and patients who receive cancer chemotherapy exhibit progressively worse physical status over time. We did not adequately examine patient confounding factors including tumor metastasis, line of treatment, progression or recurrence of disease, or employment and marital status. Third, our evaluation was limited to the effects of pharmaceutical care for peripheral neuropathy, and we did not report the effects of pharmaceutical care on malaise, edema of the limbs, pruritus, and dry skin. These reports are currently in progress. Finally, there is a lack of information regarding the degree of compliance with treatment, the possible influence of over-the-counter or complementary medicines on QOL scores, comorbidity, and treatment status.

## Conclusions

This study presented real-world data analyses showing that adverse events, such as peripheral neuropathy, malaise, and edema of the limbs, are significantly correlated with a decrease in QOL, regardless of the type of cancer or anticancer drugs used. Pharmaceutical care provided by pharmacists in collaboration with physicians may improve QOL.

## Electronic supplementary material

ESM 1 (DOCX 39 kb)
