# Supplementary material for: Real-world data of the association between quality of life using the EuroQol 5 Dimension 5 Level utility value and adverse events for outpatient cancer chemotherapy
Source: Support Care Cancer. 2020 Apr 12;28(12):5943–52. doi: 10.1007/s00520-020-05443-8 (PMC7686000; doi:10.1007/s00520-020-05443-8)
Supplement: Supplementary file 1 — (DOCX 39 kb) [file 520_2020_5443_MOESM1_ESM.docx]

Supplemental Table 1. The EQ5D utility value and 5 dimensions of the EuroQol 5 Dimension 5 Level questionnaire of patients with different cancer types, under different regimens and with different adverse events

|  | Utility value | Mobility | | | | | Personal care | | | | | Usual activities | | | | | Pain/discomfort | | | | | Anxiety/depression | | | | |
| --- | --- | --- | --- | --- | --- | --- | --- | --- | --- | --- | --- | --- | --- | --- | --- | --- | --- | --- | --- | --- | --- | --- | --- | --- | --- | --- |
|  |  | 1 | 2 | 3 | 4 | 5 | 1 | 2 | 3 | 4 | 5 | 1 | 2 | 3 | 4 | 5 | 1 | 2 | 3 | 4 | 5 | 1 | 2 | 3 | 4 | 5 |
| Male (2369) | 0.828 ± 0.138 | 66% | 25% | 5% | 3% | 0% | 85% | 11% | 2% | 1% | 0% | 65% | 26% | 5% | 3% | 0% | 52% | 39% | 6% | 3% | 0% | 69% | 25% | 3% | 1% | 0% |
| Female (2326) | 0.825 ± 0.116 | 68% | 27% | 3% | 1% | 0% | 87% | 11% | 1% | 0% | 0% | 67% | 28% | 3% | 1% | 0% | 46% | 45% | 6% | 2% | 0% | 55% | 39% | 5% | 1% | 0% |
| Age ≥65 (2551) | 0.821 ± 0.138 | 62% | 28% | 6% | 3% | 0% | 84% | 11% | 3% | 1% | 0% | 64% | 26% | 5% | 3% | 1% | 52% | 38% | 6% | 3% | 0% | 65% | 28% | 4% | 1% | 0% |
| Age <65 (2144) | 0.833 ± 0.113 | 74% | 23% | 2% | 1% | 0% | 88% | 10% | 1% | 0% | 0% | 68% | 28% | 3% | 1% | 0% | 46% | 46% | 5% | 2% | 0% | 58% | 36% | 4% | 1% | 0% |
| Cancer type (n) |  |  |  |  |  |  |  |  |  |  |  |  |  |  |  |  |  |  |  |  |  |  |  |  |  |  |
| Pancreatic cancer (590) | 0.802± 0.140 | 55% | 37% | 6% | 2% | 0% | 80% | 15% | 3% | 1% | 0% | 58% | 32% | 7% | 2% | 0% | 42% | 47% | 8% | 2% | 0% | 57% | 35% | 5% | 3% | 0% |
| Gastric cancer (656) | 0.807± 0.151 | 64% | 26% | 5% | 5% | 0% | 82% | 13% | 3% | 2% | 0% | 59% | 29% | 5% | 5% | 0% | 46% | 41% | 8% | 4% | 0% | 58% | 34% | 6% | 1% | 0% |
| Esophageal cancer (60) | 0.871± 0.082 | 78% | 18% | 2% | 0% | 0% | 95% | 3% | 0% | 0% | 0% | 80% | 17% | 2% | 0% | 0% | 45% | 43% | 10% | 0% | 0% | 78% | 18% | 2% | 0% | 0% |
| Head and neck cancer (70) | 0.828± 0.116 | 74% | 24% | 0% | 1% | 0% | 94% | 6% | 0% | 0% | 0% | 50% | 49% | 0% | 1% | 0% | 44% | 50% | 3% | 3% | 0% | 59% | 40% | 0% | 1% | 0% |
| Colorectal cancer (731) | 0.840± 0.110 | 70% | 25% | 3% | 1% | 0% | 88% | 11% | 1% | 0% | 0% | 68% | 28% | 2% | 2% | 0% | 53% | 40% | 4% | 3% | 0% | 67% | 29% | 2% | 1% | 1% |
| Lung cancer (406) | 0.840± 0.113 | 69% | 24% | 4% | 1% | 1% | 91% | 5% | 2% | 1% | 0% | 71% | 22% | 5% | 0% | 1% | 56% | 39% | 3% | 0% | 0% | 63% | 32% | 3% | 0% | 0% |
| Anticancer drugs (n) |  |  |  |  |  |  |  |  |  |  |  |  |  |  |  |  |  |  |  |  |  |  |  |  |  |  |
| Oxliplatin (726) | 0.827± 0.124 | 72% | 23% | 3% | 2% | 0% | 85% | 12% | 2% | 1% | 0% | 62% | 32% | 3% | 2% | 0% | 48% | 44% | 6% | 2% | 0% | 61% | 33% | 4% | 1% | 1% |
| Paclitaxel (1259) | 0.825± 0.127 | 66% | 27% | 4% | 2% | 0% | 86% | 10% | 2% | 1% | 0% | 65% | 28% | 4% | 2% | 0% | 44% | 45% | 8% | 3% | 0% | 63% | 32% | 4% | 1% | 0% |
| Irinotecan (529) | 0.817± 0.111 | 59% | 39% | 2% | 1% | 0% | 85% | 14% | 1% | 0% | 0% | 61% | 34% | 3% | 1% | 0% | 43% | 50% | 5% | 2% | 0% | 57% | 35% | 6% | 1% | 0% |
| Cetuximab/  panitumumab (221) | 0.842± 0.116 | 73% | 24% | 2% | 1% | 0% | 86% | 12% | 1% | 0% | 0% | 63% | 32% | 2% | 3% | 0% | 48% | 46% | 2% | 3% | 0% | 74% | 23% | 2% | 0% | 1% |
| Anthracycline + cyclophosphamide (181) | 0.839± 0.115 | 75% | 18% | 4% | 3% | 0% | 89% | 9% | 2% | 1% | 0% | 64% | 32% | 3% | 1% | 0% | 55% | 31% | 9% | 3% | 0% | 69% | 26% | 4% | 1% | 0% |
| Vincristine (113) | 0.818± 0.131 | 64% | 26% | 7% | 4% | 0% | 84% | 12% | 3% | 1% | 0% | 54% | 39% | 5% | 2% | 0% | 50% | 34% | 12% | 4% | 0% | 71% | 23% | 4% | 1% | 0% |
| Docetaxel (172) | 0.843± 0.112 | 68% | 26% | 5% | 2% | 0% | 90% | 10% | 1% | 0% | 0% | 68% | 23% | 9% | 1% | 0% | 58% | 38% | 3% | 0% | 0% | 67% | 28% | 3% | 1% | 1% |
| Carboplatin (188) | 0.820± 0.128 | 63% | 31% | 4% | 1% | 1% | 85% | 13% | 1% | 1% | 0% | 65% | 29% | 3% | 2% | 0% | 48% | 43% | 5% | 2% | 1% | 54% | 39% | 4% | 2% | 0% |
| Gemcitabine (547) | 0.813± 0.143 | 57% | 32% | 7% | 2% | 0% | 83% | 11% | 4% | 1% | 0% | 60% | 29% | 7% | 2% | 0% | 50% | 39% | 8% | 2% | 0% | 63% | 30% | 4% | 3% | 0% |
| Nivolumab/  pembrolizumab (568) | 0.826± 0.130 | 66% | 23% | 7% | 3% | 1% | 84% | 14% | 1% | 1% | 0% | 64% | 27% | 6% | 2% | 1% | 50% | 42% | 6% | 2% | 0% | 67% | 29% | 3% | 1% | 0% |
| Adverse events  (grade ≥2) (n) |  |  |  |  |  |  |  |  |  |  |  |  |  |  |  |  |  |  |  |  |  |  |  |  |  |  |
| Constipation (148) | 0.633± 0.115 | 19% | 38% | 33% | 8% | 1% | 56% | 31% | 10% | 1% | 1% | 11% | 46% | 31% | 8% | 1% | 9% | 34% | 43% | 15% | 0% | 28% | 41% | 26% | 4% | 1% |
| Malaise (86) | 0.583± 0.154 | 23% | 30% | 23% | 21% | 2% | 51% | 27% | 16% | 6% | 0% | 12% | 40% | 37% | 10% | 0% | 12% | 23% | 35% | 28% | 2% | 20% | 29% | 30% | 19% | 2% |
| Anorexia (120) | 0.611± 0.134 | 15% | 35% | 34% | 15% | 0% | 58% | 28% | 12% | 3% | 0% | 10% | 34% | 41% | 13% | 2% | 8% | 32% | 50% | 10% | 0% | 21% | 44% | 30% | 3% | 2% |
| Peripheral neuropathy (119) | 0.618± 0.136 | 11% | 39% | 36% | 13% | 0% | 46% | 31% | 19% | 3% | 0% | 13% | 51% | 25% | 8% | 1% | 5% | 32% | 43% | 18% | 1% | 32% | 34% | 26% | 7% | 0% |
| Alopecia (91) | 0.672± 0.105 | 31% | 45% | 19% | 5% | 0% | 69% | 23% | 8% | 0% | 0% | 22% | 54% | 21% | 3% | 0% | 5% | 34% | 45% | 14% | 1% | 26% | 37% | 31% | 5% | 0% |
| Nausea (40) | 0.605± 0.142 | 35% | 33% | 25% | 8% | 0% | 55% | 25% | 18% | 3% | 0% | 20% | 38% | 30% | 10% | 3% | 3% | 13% | 63% | 23% | 0% | 13% | 30% | 35% | 23% | 0% |
| Taste disorder (42) | 0.632± 0.117 | 12% | 43% | 33% | 12% | 0% | 64% | 31% | 5% | 0% | 0% | 7% | 52% | 31% | 10% | 0% | 5% | 43% | 38% | 14% | 0% | 26% | 55% | 10% | 7% | 2% |
| Utility values are represented as mean ± standard deviation. Values for the 5 dimensions indicate percentage of each item. | | | | | | | | | | | | | | | | | | | | | | | | | | |

Supplemental Table 2. Demographics of patients who received intervention by a pharmacist for peripheral neuropathy

| Number of patients (male/female) | 36 | (16/20) |
| --- | --- | --- |
| Age, median (min–max) | 62 | (39-81) |
| Cancer |  |  |
| Colorectal cancer | 8 | 22.2% |
| Gastric cancer | 7 | 19.4% |
| Pancreatic cancer | 13 | 36.1% |
| Breast cancer | 5 | 13.9% |
| Other | 3 | 8.3% |
| Regimen |  |  |
| Weekly PTX/Nab-PTX ± trastuzumab/ramcirumab | 17 | 47.2% |
| CapeOX/SOX/FOLFOX ± bevacuzumab/cetuximab/panitumumab | 6 | 16.7% |
| FOLFIRINOX/FOLFOXIRI ± bevacuzumab/cetuximab | 8 | 22.2% |
| Other | 5 | 13.9% |
| Details of intervention |  |  |
| Additional oral administration of pregabalin | 13 | 36.1% |
| Additional oral administration of duloxetine | 20 | 55.6% |
| Additional oral administration of goshajinkigan | 1 | 2.8% |
| Cooling for the hands and feet | 2 | 5.6% |
| All data indicate median, 25-75^th^ percentiles unless otherwise indicated | | |
